# Correction: MS-ConTab: multi-scale contrastive learning of mutation signatures for Pan-Cancer representation and stratification

**DOI:** 10.1093/bioinformatics/btag650

**Published:** 2026-09-02

This is a correction to: Yifan Dou, Adam Khadre, Ruben C Petreaca, Golrokh Mirzaei, MS-ConTab: multi-scale contrastive learning of mutation signatures for Pan-Cancer representation and stratification, *Bioinformatics*, Volume 42, Issue 5, May 2026, https://doi.org/10.1093/bioinformatics/btag131

The following change has been made to the originally published paper.

The first and last names of the fourth co-author were erroneously transposed. These have been corrected to read: **“Golrokh Mirzaei”**.

