# Supplementary material for: MS-ConTab: multi-scale contrastive learning of mutation signatures for Pan-Cancer representation and stratification
Source: Bioinformatics. 2026 Apr 27;42(5):btag131. doi: 10.1093/bioinformatics/btag131 (PMC13139774; doi:10.1093/bioinformatics/btag131)

**Supplementary Table 1: Nearest neighbor analysis of cancer-type embeddings learned by MS-ConTab**. For each of the 42 cancer types, the three most similar cancer types are listed based on cosine similarity in the learned embedding space. This analysis highlights biologically related groupings as well as cross-lineage proximities revealed by the model.

| **Nearest Neighbors for Each Cancer Type:** |
| --- |
| Adrenal gland : ['Cervix', 'Ovary', 'Pancreas']  Autonomic ganglia : ['NS', 'Breast', 'Fallopian tube']  Biliary tract : ['Thymus', 'Female Genital Tract', 'Haemathopoietic and Lymphoid']  Bone : ['Ovary', 'Endometrium', 'Pancreas']  Breast : ['Salivary gland', 'Pleura', 'Testis']  CNS : ['Liver', 'Thyroid', 'Eye']  Cervix : ['Pancreas', 'Endometrium', 'Ovary']  Endometrium : ['Bone', 'Ovary', 'Pancreas']  Eye : ['Thyroid', 'Lung', 'Large intestine']  Fallopian tube : ['Penis', 'Placenta', 'Pleura']  Female Genital Tract : ['Uterine adnexa', 'Vulva', 'Vagina']  Gastrointestinal tract : ['Uterine adnexa', 'Vagina', 'Vulva']  Genital Tract : ['Parathyroid', 'Vagina', 'Gastrointestinal tract']  Haemathopoietic and Lymphoid: ['Testis', 'Pleura', 'Thymus']  Kidney : ['Meninges', 'Skin', 'Up Aero']  Large intestine : ['Prostate', 'Lung', 'Pancreas']  Liver : ['CNS', 'Thyroid', 'Up Aero']  Lung : ['Large intestine', 'Eye', 'Thyroid']  Meninges : ['Kidney', 'Skin', 'Up Aero']  NS : ['Autonomic ganglia', 'Breast', 'Fallopian tube']  Oesophagus : ['Endometrium', 'Ovary', 'Prostate']  Ovary : ['Bone', 'Endometrium', 'Pancreas']  Pancreas : ['Bone', 'Ovary', 'Endometrium']  Parathyroid : ['Genital Tract', 'Vagina', 'Gastrointestinal tract']  Penis : ['Testis', 'Placenta', 'Pleura']  Peritoneum : ['Vulva', 'Uterine adnexa', 'Gastrointestinal tract']  Pituitary : ['Parathyroid', 'Genital Tract', 'Gastrointestinal tract']  Placenta : ['Testis', 'Pleura', 'Haemathopoietic and Lymphoid']  Pleura : ['Testis', 'Haemathopoietic and Lymphoid', 'Placenta']  Prostate : ['Oesophagus', 'Ovary', 'Endometrium']  Salivary gland : ['Pleura', 'Penis', 'Testis']  Skin : ['Stomach', 'Kidney', 'Meninges']  Small intestine : ['Pancreas', 'Cervix', 'Bone']  Soft tissue : ['Pancreas', 'Ovary', 'Endometrium']  Stomach : ['Skin', 'Meninges', 'Liver']  Testis : ['Pleura', 'Haemathopoietic and Lymphoid', 'Placenta']  Thymus : ['Female Genital Tract', 'Vulva', 'Haemathopoietic and Lymphoid']  Thyroid : ['Eye', 'Lung', 'Liver']  Up Aero : ['Meninges', 'Liver', 'Kidney']  Urinary : ['Oesophagus', 'Endometrium', 'Bone']  Uterine adnexa : ['Gastrointestinal tract', 'Vulva', 'Vagina']  Vagina : ['Gastrointestinal tract', 'Uterine adnexa', 'Vulva']  Vulva : ['Gastrointestinal tract', 'Uterine adnexa', 'Vagina'] |

**Supplementary Table 2. Shared and cluster-specific mutated genes across cancer clusters.**This table lists the genes found to be mutated in each cluster, stratified into those shared between both clusters (n = 117) and those unique to Cluster 1 (n = 73) or Cluster 2 (n = 148). Shared genes include key pan-cancer drivers (e.g., TP53, KRAS, PIK3CA, PTEN), whereas Cluster 1-specific genes are enriched for epithelial and structural regulators (e.g., FOXL2, STK11, KEAP1), and Cluster 2-specific genes are enriched for hematopoietic and transcriptional regulators (e.g., JAK2, RUNX1, MYC, EZH2).

| **Genes common to both clusters:** |
| --- |
| ['ABCA13', 'ADGRV1', 'AHNAK', 'AHNAK2', 'ALK', 'ANK3', 'ANKRD36C', 'APC', 'APOB', 'AR', 'ARID1A', 'ATM', 'ATR', 'ATRX', 'BAP1', 'BRAF', 'BRCA1', 'BRCA2', 'CDKN2A', 'CHEK2', 'CMYA5', 'CREBBP', 'CSMD1', 'CSMD2', 'CSMD3', 'CTNNB1', 'CYLD', 'DCC', 'DICER1', 'DMD', 'DNAH5', 'DNAH7', 'DNAH8', 'EGFR', 'EIF1AX', 'EP300', 'EPAS1', 'ERBB2', 'ERBB3', 'FAT1', 'FAT3', 'FAT4', 'FBXW7', 'FGFR3', 'FLG', 'GLI3', 'GNA11', 'GNAS', 'GRIN2A', 'GTF2I', 'HLA-A', 'HMCN1', 'HNRNPCL1', 'HRAS', 'IDH1', 'IDH2', 'KCNJ12', 'KDM6A', 'KIT', 'KMT2A', 'KMT2C', 'KMT2D', 'KRAS', 'LRP1B', 'MACF1', 'MED12', 'MEN1', 'MET', 'MSH6', 'MTOR', 'MUC12', 'MUC16', 'MUC17', 'MUC4', 'MUC5B', 'MUC6', 'NBPF1', 'NBPF10', 'NEB', 'NF1', 'NFE2L2', 'NOTCH1', 'NOTCH2', 'NRAS', 'OBSCN', 'PABPC1', 'PABPC3', 'PBRM1', 'PCLO', 'PDGFRA', 'PIK3CA', 'POLE', 'PPP2R1A', 'PRAMEF10', 'PRKDC', 'PTCH1', 'PTEN', 'PTPRT', 'RB1', 'RET', 'RYR1', 'RYR2', 'RYR3', 'SETD2', 'SF3B1', 'SMAD4', 'SMARCA4', 'SYNE1', 'TAS2R30', 'TET2', 'TP53', 'TSC2', 'TTN', 'USH2A', 'WT1', 'ZFHX3', 'ZFHX4'] |
| **Genes unique to Cluster 1:** |
| ['ABCA7', 'AC058822.1', 'ACAN', 'ADAMTS7', 'AKAP13', 'ALPK2', 'ANKRD36', 'APOBR', 'ATP1A1', 'BICRA', 'BLM', 'CACNA1D', 'CDC27', 'CDK12', 'CENPF', 'CIC', 'COL2A1', 'DNAH3', 'DROSHA', 'ERCC2', 'FAM135B', 'FLG2', 'FOXL2', 'GNAQ', 'GNL3', 'H3F3A', 'H3F3B', 'HELZ2', 'HERC2', 'HLA-B', 'HLA-DRB1', 'HNF1A', 'HRNR', 'HSPA1L', 'HYDIN', 'IGFN1', 'JAK1', 'KCNJ5', 'KDM5C', 'KEAP1', 'LRP2', 'MAGEA12', 'MKI67', 'MUC3A', 'MYO15B', 'NAV3', 'NBEA', 'OR1S1', 'OR1S2', 'OR4C3', 'OR4C5', 'OR8U1', 'PDE4DIP', 'PKHD1', 'PKHD1L1', 'PLEC', 'POTEC', 'PRB2', 'PRKACA', 'RFPL4AL1', 'RGPD3', 'RNF213', 'RNF43', 'SACS', 'SCN9A', 'SLC25A5', 'SPOP', 'SPTA1', 'SSPO', 'STAG2', 'STK11', 'SVEP1', 'SYNE2', 'VHL', 'XIRP2', 'ZBTB7A', 'ZNF429', 'ZNF717', 'ZSCAN5A'] |
| **Genes unique to Cluster 2:** |
| ['ABCB4', 'ACAD9', 'ACSM5', 'ADAM7', 'ADGRL2', 'AKAP9', 'AKT1', 'ARID1B', 'ARSD', 'BCL2', 'BCL9', 'BCLAF1', 'BEND5', 'BIRC6', 'BIVM-ERCC5', 'CACNA1E', 'CBL', 'CDC73', 'CDH1', 'CDKN1B', 'CDKN2C', 'CLASP2', 'COL7A1', 'CRLF2', 'CXCR4', 'DDR2', 'DDX3X', 'DHX38', 'DHX9', 'DNAH2', 'DNMT3A', 'DPYSL2', 'EPRS', 'ERBB4', 'ERCC5', 'ERCC6', 'ESR1', 'ESRRA', 'EXOC2', 'EZH2', 'FGD6', 'FGFR2', 'FLT3', 'FLT4', 'FOXA1', 'FSIP2', 'GATA3', 'GLUD2', 'GOT1L1', 'HLA-DQB1', 'HNRNPA2B1', 'HS6ST3', 'HSPA5', 'IL6ST', 'INTS1', 'ITPRID2', 'JAK2', 'KCND1', 'KDR', 'KIAA0586', 'KRTAP4-8', 'LATS2', 'LDLR', 'LILRA2', 'LILRB2', 'LYST', 'MAP2', 'MAP2K3', 'MAP3K1', 'MLH1', 'MSH2', 'MSH3', 'MUCL3', 'MXRA5', 'MYC', 'MYCN', 'MYD88', 'MYH1', 'MYH9', 'NCOR1', 'NCOR2', 'NF2', 'NFAT5', 'NFKBIA', 'NFXL1', 'NOTCH4', 'NRG1', 'NSD1', 'NTRK1', 'NTRK2', 'NTRK3', 'NUGGC', 'NUP214', 'OR56A5', 'OR5D13', 'OR5M1', 'OR7C1', 'OSBPL1A', 'P2RY14', 'PAX3', 'PDE7A', 'PDGFRB', 'PDZD2', 'PHF20L1', 'PIK3R1', 'PIK3R2', 'PIM1', 'PLA2G4C', 'PLXNA2', 'PMM1', 'PMS2', 'POLR1A', 'PRDM8', 'PRKCG', 'PRKD1', 'PRUNE2', 'PTK2B', 'PTPN11', 'PTPN7', 'PTPRB', 'PTPRD', 'PTPRS', 'QRICH2', 'RAC1', 'RAF1', 'RASAL1', 'RASGEF1B', 'RBM41', 'RECQL4', 'RGS16', 'RGS5', 'RIF1', 'ROCK2', 'ROS1', 'RP1', 'RRAS2', 'RRP7A', 'RUNX1', 'SAA1', 'SERPINA12', 'SETD1B', 'SETDB1', 'SETX', 'SH2B3', 'SMAD2', 'SMARCA5', 'SMO', 'SOCS1', 'SPEN', 'SRSF2', 'STARD9', 'SUPT5H', 'TAF1L', 'TLN1', 'TMTC3', 'TPSD1', 'TRAF7', 'UBC', 'UBE4B', 'USP28', 'USP6', 'USP8', 'VANGL2', 'VMP1', 'VPS13B', 'WDR43', 'WSCD2', 'ZBTB8A', 'ZEB1', 'ZFP62', 'ZNF117', 'ZNF430', 'ZNF443', 'ZNF708'] |

**Supplementary Table 3. Comparison of clustering performance for different embedding approaches using k-means (k=2).** Higher Silhouette and Calinski–Harabasz (CH) indices and lower Davies–Bouldin (DB) indices indicate better clustering quality. Metrics are reported both in the original embedding space and after UMAP projection (for visualization consistency).

| **Embedding** | **Space** | **Silhouette Score (↑)** | **DB Index (↓)** | **CH Index (↑)** |
| --- | --- | --- | --- | --- |
| TABNet+Constrastive | Original | 0.561 | 0.655 | 43.106 |
|  | UMAP | 0.906 | 0.136 | 1862.023 |
| NMF (k=43) | Original | 0.141 | 3.011 | 2.680 |
|  | UMAP | 0.466 | 1.503 | 15.547 |
| Hierarchical (Ward) | original | 0.697 | 0.194 | 16.275 |
|  | UMAP | 0.467 | 0.755 | 58.403 |
| Autoencoder (AE) | Original | 0.187 | 1.866 | 2.385 |
|  | UMAP | 0.562 | 0.586 | 99.993 |
| SimCLR (MLP-based) | Original | 0.239 | 1.378 | 4.721 |
|  | UMAP | 0.600 | 0.532 | 119.691 |

**Supplementary Figure 1. Gene and chromosome level analysis data parsing. A**) Data filtering for subsequent data analysis. COSMIC reports both data for the dominant transcript of each gene as well as possible alternative transcripts. The dominant transcript is labeled with the gene name (e.g TP53) while the alternative transcripts are labeled with gene name followed by transcript identifier (e.g. TP53ENST_0235). We first removed all alternative transcript data, then removed all non-coding data (5’UTR, 3’UTR and intronic). Nucleotide substitutions were then extracted for protein coding regions (exons). **B**) Gene and Chromosome level data analysis. Gene-level analysis was performed using the counts of each nucleotide substitution per gene. For chromosome-level analysis, substitution counts were normalized by chromosome length, calculated by dividing the number of substitutions by the corresponding chromosome’s length. This normalization allowed for accurate comparisons across chromosomes of varying sizes. **C**) Possible nucleotide substitutions at each locus. For this analysis we assumed that each nucleotide could be substituted to the three different ones.


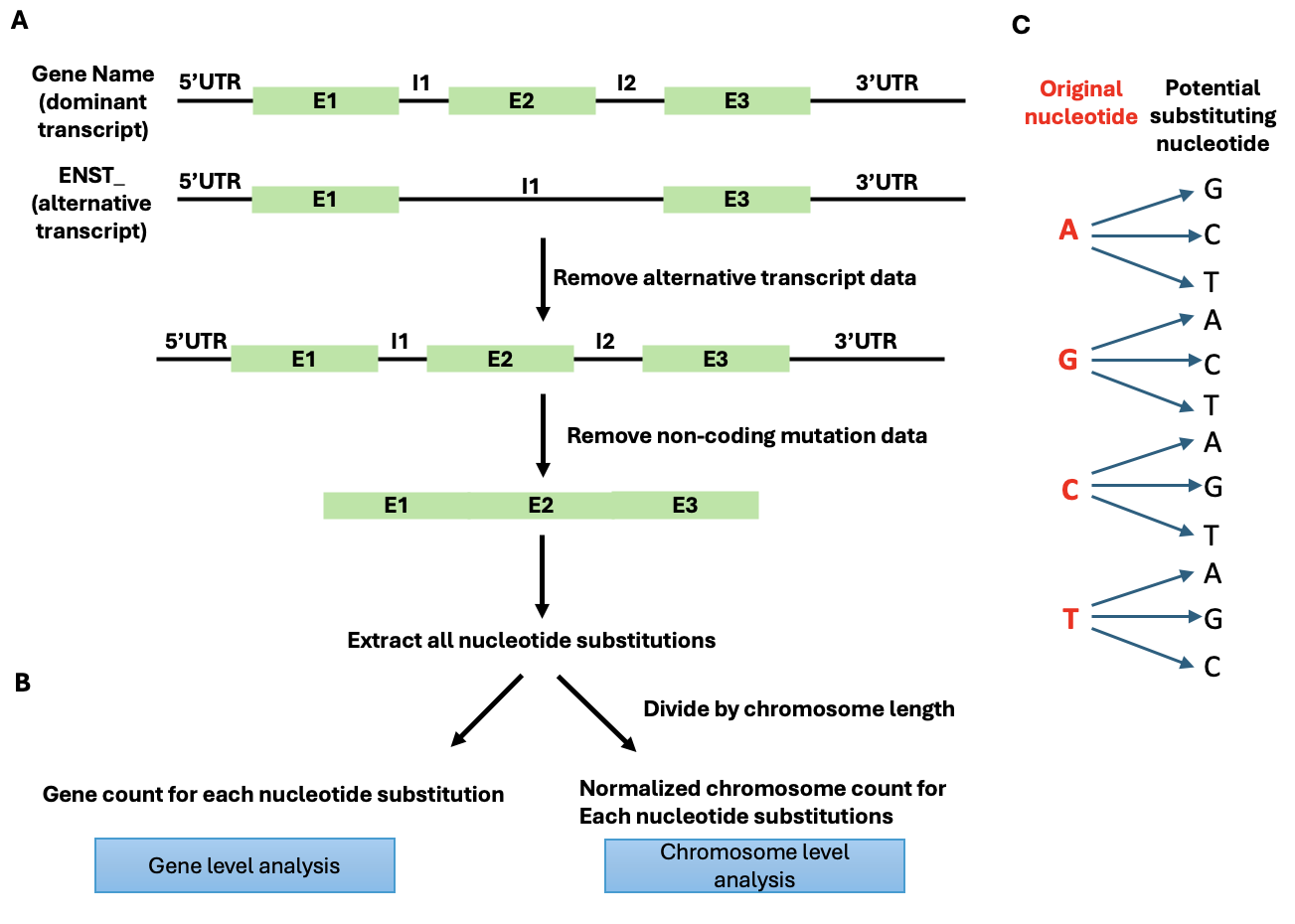

Supplement: btag131_Supplementary_Data [file btag131_supplementary_data.zip › Supplementary final.docx]
